# Future orientation moderates the relationship between medication adherence and health-related quality of life among people with drug-resistant tuberculosis in Lagos, Nigeria: a cross-sectional study

**DOI:** 10.1186/s12879-026-14163-z

**Published:** 2026-08-06

**Authors:** Olusola Adedeji Adejumo, Champaklal Jinabhai, Firoza Haffejee

**Affiliations:** 1 Department of Basic Medical Sciences, Durban University of Technology, Durban, South Africa; 2 Mainland Hospital, Yaba, Lagos, Nigeria; 3 Faculty of Health Sciences, Durban University of Technology, Durban, South Africa

**Keywords:** Future orientation, Health-related quality of life, Medication adherence, Drug-resistant tuberculosis

## Abstract

**Background:**

Future orientation is the tendency to prioritise long-term outcomes over immediate consequences, which may influence how individuals engage with prolonged and demanding treatment regimens. Its role in shaping patient-centred outcomes among people with drug-resistant tuberculosis (PwDR-TB) remains poorly understood. This study examined whether future orientation moderates the association between medication adherence and health-related quality of life (HRQoL) among PwDR-TB in Lagos, Nigeria.

**Methods:**

A cross-sectional study was conducted among 203 PwDR-TB receiving care at five treatment centres in Lagos. HRQoL was assessed with the Functional Assessment of Chronic Illness Therapy–Tuberculosis (FACIT-TB), medication adherence with the 8-item Morisky Medication Adherence Scale (MMAS-8), and future orientation with the Deferment of Gratification Scale. Hierarchical multiple regression was used to examine the moderating effect of future orientation on the association between adherence and HRQoL, adjusting for relevant sociodemographic and clinical covariates. Simple slopes analysis was conducted to interpret significant interactions.

**Results:**

Participants classified as adherent had significantly higher HRQoL scores across all domains (*p* < 0.001). Medication adherence was positively associated with HRQoL (β = 0.316, *p* < 0.001), as was future orientation (β = 0.262, *p* < 0.001). The interaction between adherence and future orientation was statistically significant (β = 0.201, *p* = 0.018), indicating moderation. However, the interaction explained a small proportion of additional variance in HRQoL (ΔR² = 0.016), suggesting a modest effect size. The positive association between adherence and HRQoL was stronger at higher levels of future orientation.

**Conclusion:**

Future orientation was associated with differences in the relationship between adherence and HRQoL, although the interaction was modest. Longitudinal studies are required to determine whether future orientation prospectively modifies this relationship before implications for intervention can be established.

**Supplementary Information:**

The online version contains supplementary material available at https://doi.org/10.1186/s12879-026-14163-z.

## Introduction

Tuberculosis (TB) remains the leading cause of death from a single infectious agent globally, and the increasing burden of drug-resistant tuberculosis (DR-TB) continues to undermine progress in TB control [1]. In 2024, 164,525 (42%) of the estimated 390,000 individuals who developed rifampicin-resistant or multidrug-resistant TB (MDR-TB) worldwide were treated [1]. Despite advances in diagnostics and treatment, DR-TB management remains complex, requiring prolonged therapy, a substantial pill burden, and exposure to potentially severe adverse effects. These challenges impose considerable physical, psychological, and socioeconomic strain on patients, particularly in high-burden settings such as Nigeria [2].

Medication adherence is a critical determinant of treatment success in DR-TB [3, 4]. Suboptimal adherence is associated with poor clinical outcomes, ongoing transmission, and the amplification of drug resistance [5]. However, maintaining consistent adherence over treatment durations of 9–20 months is difficult for many patients. Adherence behaviour is shaped by a complex interplay of structural, social, and psychological factors, including financial constraints, health system barriers, stigma, and individual motivation [5].

Health-related quality of life (HRQoL) has emerged as an important patient-centred outcome in TB care, reflecting individuals’ perceptions of their physical, psychological, and social well-being. Evidence consistently indicates that DR-TB substantially impairs HRQoL due to prolonged treatment, adverse drug reactions, stigma, economic hardship, and social disruption [6–8]. While medication adherence has been associated with better HRQoL in some studies [9, 10], low HRQoL may also reduce motivation to adhere to treatment [11]. Findings across studies remain inconsistent, suggesting that additional behavioural and cognitive factors may influence this relationship [11, 12].

Future orientation is a psychological construct reflecting the extent to which individuals prioritise long-term outcomes over immediate costs or rewards [13]. Rooted in behavioural economics and time-perspective theory, it has been linked to engagement in health-promoting behaviours, including medication adherence. Individuals with a stronger future orientation are more likely to tolerate short-term discomfort in pursuit of long-term benefits. In contrast, those with a present-oriented perspective may prioritise immediate relief, thereby undermining adherence [13].

The management of DR-TB requires sustained engagement with treatment despite substantial short-term burdens, including side effects, adverse drug reactions, medication toxicity, and social disruption, making future orientation particularly relevant in this context [14]. Future orientation has been associated with better medication adherence and improved health outcomes in chronic disease populations [15]. However, little is known about whether it modifies the association between medication adherence and broader patient-centred outcomes, such as HRQoL [16]. Understanding this relationship is especially important in high-burden settings such as Nigeria, where socioeconomic constraints, health system challenges, and treatment-related burdens may further complicate sustained engagement with long-term DR-TB treatment [17–19].

This study addresses this gap by examining whether future orientation moderates the association between medication adherence and HRQoL among people receiving treatment for DR-TB in Lagos, Nigeria. We hypothesised that medication adherence would be positively associated with HRQoL and that this association would be stronger among individuals with higher levels of future orientation.

## Methods

### Study design

We conducted an analytical cross-sectional study to assess the moderating effects of future orientation on the association between medication adherence and HRQoL among PwDR-TB in Lagos, Nigeria. The analyses examined cross-sectional associations and statistical interaction rather than temporal or causal moderation because data were collected at a single time point,

### Study setting and programmatic management of DRTB

Lagos is a cosmopolitan city in Nigeria, with an estimated population of about 21 million (about 11% of Nigeria’s population). Under the Lagos State TB, Leprosy and Buruli ulcer control programme, Xpert MTB/RIF assay is used for DR-TB diagnosis and newly diagnosed patients are contacted via SMS by the state TB programme. Treatment initiation took place at six designated health facilities where patients received education on DR-TB transmission, treatment, side effects, and available support services. Following baseline laboratory investigations, patients were referred to the nearest treatment centre for routine nurse-led treatment monitoring and follow-up as part of standard care. Transportation costs were reimbursed to facilitate clinic attendance and support adherence throughout treatment.

The recommended treatment for DR-TB in Nigeria is the 6-month BPaLM/BPaL regimen for patients aged 14 years and older with MDR-TB, which was introduced nationally in November 2023. In mid-2025, the 6-month BDLLfxC/BDLLfx/BDLC regimen was adopted for children younger than 14 years, pregnant and breastfeeding women, with regimen selection guided by drug-susceptibility test (DST) results and the patient’s history of prior drug exposure or intolerance. Before this update, the World Health Organization (WHO) recommended a shorter all-oral regimen for pregnant and breastfeeding women because the safety of pretomanid in these populations had not been established. Patients with osteoarticular, central nervous system (CNS), or disseminated DR-TB are treated with a longer 18-month regimen (6Bdq-Mfx-Lzd-Cfz/12Mfx-Cfz-Lzd) owing to the complexity and severity of these disease manifestations. Individualised treatment regimens are reserved for extensively drug-resistant tuberculosis (XDR-TB) and cases with confirmed resistance to either bedaquiline or linezolid. These consist of 6Dlm-Lzd-Cfz-Cs-Z/12Lzd-Cfz-Cs-Z for bedaquiline-resistant disease or 6Bdq-Dlm-Cfz-Cs-Z/12Dlm-Cfz-Cs for linezolid-resistant disease.

### Selection of study sites and participants

Of the six DR-TB treatment centres in Lagos, five with the highest patient volumes were purposively selected to ensure broad geographic representation and a diverse study population. This study was conducted as part of a broader research project investigating the relationships among stigma, anxiety, depression, loneliness, and social support among PwDR-TB in Lagos [20]. A total of 203 participants were consecutively recruited following their outpatient clinic visits. The sample size determination was earlier published [20]. Eligibility criteria included adults aged 18 years and above who had been on treatment for at least eight weeks and were able to understand English. Patients who were severely ill and unable to provide informed consent due to cognitive impairment were excluded from the study.

### Measures

In this study, HRQoL is the outcome measure, while the independent variables are medication adherence and future orientation. Additional covariates included age, sex, marital status, HIV status, facility type, perceived severity of DR-TB, perceptions of DR-TB treatment, tuberculosis knowledge, duration of treatment, income, cigarette smoking, and alcohol consumption. An interaction term was constructed as a composite variable representing the product of medication adherence and future orientation.

### Measurement instruments

The Functional Assessment of Chronic Illness Therapy–Tuberculosis (FACIT-TB) tool was used to assess the HRQoL in this study [21]. It is a 47-item scale that comprises five domains: physical, social, emotional, functional, and spiritual well-being. Total scores for the FACIT-TB scale range from 0 to 188, with higher scores indicating better health status and functional well-being. To allow comparability across domains with differing item counts, domain scores were transformed to a 0–100 scale. Subscale scores were presented as domain-specific profiles, and the overall HRQoL score was calculated by summing all subscale transformed scores. The FACIT-TB demonstrated good internal consistency in this study, with a Cronbach’s alpha of 0.825. It is a validated TB-specific multidimensional quality-of-life instrument used in a previous study [21].

Future orientation was assessed using the Deferment of Gratification Scale (DGS) [22]. This 12-item validated instrument measures an individual’s ability to endure immediate discomfort in pursuit of longer-term rewards. It includes six positively worded items and six reverse-scored items, with responses recorded on a 7-point Likert scale (1 = strongly disagree to 7 = strongly agree) [22]. Higher total scores indicate a stronger preference for delayed gratification, whereas lower scores reflect greater overlooking of future benefits (i.e., a stronger preference for immediate rewards). In the present study, the DGS exhibited acceptable internal consistency (Cronbach’s α = 0.780).

Medication adherence was measured using the 8-item Morisky Medication Adherence Scale (MMAS-8) [23]. The first seven items are dichotomous (yes/no), while the eighth item assesses the frequency of non-adherence using a 5-point Likert scale [24]. The MMAS-8 captures both unintentional and intentional non-adherence behaviours. Total scores range from 0 to 8, with scores < 6 indicating low adherence, 6 to < 8 indicating medium adherence, and 8 indicating high adherence. In this study, scores < 6 were classified as non-adherence, and scores ≥ 6 as adherence [3]. The MMAS-8 demonstrated good internal reliability in this study (Cronbach’s α = 0.809).

Knowledge of tuberculosis was assessed using an 8-item questionnaire covering key aspects of TB transmission, prevention, and control. The items were developed based on a review of the relevant literature [25, 26]. Each correct response was awarded one point, while incorrect or “unsure” responses received zero points. The median score of 5 was used as the cutoff for categorization. Participants with scores below the median were classified as having poor TB knowledge, whereas those with scores of 5 or higher were classified as having good TB knowledge [26].

### Pilot testing

The study instruments were piloted with 20 volunteers at a DR-TB treatment facility from which participants were not recruited for the main study. Internal consistency was assessed using Cronbach’s alpha. Following the pilot testing, selected questionnaire items were reworded to improve clarity, validity, and acceptability.

### Data collection

Data were collected by five research assistants who received prior training in the study objectives, ethical considerations, and the administration of the data-collection instruments. The research assistants also participated in the pilot study, which ensured familiarity with the tools and standardised data collection procedures across study sites.

### Ethical considerations

Ethical approval for the study was obtained from the Institutional Research Ethics Committee of Durban University of Technology, South Africa (IREC 066/230), and the Health Research and Ethics Committee of Lagos State University Teaching Hospital, Nigeria (LREC/06/10/2179). Permission to conduct interviews was also granted by the Lagos State Ministry of Health and the Medical Directors of all participating health facilities. Written informed consent was obtained from all participants before enrolment, and all data were collected anonymously and handled confidentially throughout the study.

### Data analysis

Completed questionnaires were reviewed by the research assistants for completeness and accuracy before data entry into IBM SPSS Statistics Version 26 (IBM Corp., Armonk, NY, USA). Continuous variables were assessed for normality using the Shapiro–Wilk test and visual inspection of the distributional plots and were reported as medians and interquartile ranges. Categorical variables were summarised using frequencies and proportions. Mann-Whitney U and Kruskal-Wallis tests were used to compare total HRQoL across demographic and clinical groups.

Partial correlation analyses were conducted to evaluate bivariate associations between medication adherence and HRQoL while controlling for future orientation. Hierarchical multiple regression was used to assess the moderating role of future orientation in the association between medication adherence and HRQoL. Assumptions of normality, linearity, multicollinearity (variance inflation factor < 10; tolerance > 0.1), and homoscedasticity were verified and met.

To evaluate whether future orientation moderates the relationship between medication adherence and HRQoL, a hierarchical linear regression (for continuous adherence scores) was employed. In the hierarchical regression, Step 1, covariates significant in the bivariate analyses (age, gender, marital status, educational status and HIV status) were entered as control variables. Step 2: The main effects of medication adherence and future orientation were added, and in Step 3, the interaction term (medication adherence and future orientation) was introduced to test the moderation hypothesis. Medication adherence and future orientation were mean-centred before creating interaction terms to reduce multicollinearity. Simple slopes analysis was conducted. Predicted HRQoL values were plotted at low (− 1 SD), mean, and high (+ 1 SD) levels of future orientation to facilitate interpretation of its moderating effect. Two-sided tests were used for all analyses, with 95% confidence intervals, and *P* < 0.05 was considered statistically significant.

## Results

A total of 203 PwDR-TB were included in the study. The median age was 37 years (IQR 28–47), 75 (36.9%) were female, approximately half (49.8%) had secondary education, and 35 (17.2%) were HIV-positive. Most participants (181, 89.2%) demonstrated good knowledge of TB, while 36 (17.7%) reported barriers to accessing TB treatment (Table 1).

**Table 1:** Factors associated with total HRQoL

Variable	n (%)	Median (IQR) HRQoL	p
**Age group (years)**			
<30	54 (26.6)	42.8 (34.2, 58.5)	0.015
30–59	128 (63.1)	39.7 (32.2, 45.6)	
≥60	21 (10.3)	37.2 (30.6, 41.7)	
Median (IQR)	37 (28, 47)	—	
Range	18–80	—	
**Gender**			
Male	128 (63.1)	41.7 (33.9, 46.7)	0.025
Female	75 (36.9)	37.8 (30.6, 44.4)	
**Marital status**			
Single	74 (36.5)	41.7 (33.8, 56.0)	<0.001
Married	111 (54.7)	40.6 (33.3, 44.4)	
Widowed/Separated	18 (8.9)	30.6 (26.4, 38.5)	
**Educational status**			
No education/Primary	43 (21.2)	38.3 (25.5, 43.3)	0.003
Secondary	101 (49.8)	40.0 (33.1, 43.9)	
Tertiary	59 (29.1)	43.9 (33.9, 60.6)	
**HIV status**			
Positive	35 (17.2)	36.7 (26.7, 41.7)	0.007
Negative	160 (78.8)	41.1 (33.3, 46.7)	
Not known	8 (3.9)	36.4 (29.9, 46.4)	
**Treatment helpful**			
Yes	178 (87.7)	40.0 (32.8, 46.1)	0.843
Not sure	25 (12.3)	41.1 (29.7, 49.2)	
**Employment status**			
Employed	104 (51.2)	40.6 (34.4, 50.0)	0.065
Unemployed	99 (48.8)	39.2 (31.7, 44.4)	
**Treatment facility**			
Secondary	151 (74.4)	40.6 (32.8, 46.7)	0.461
Primary	52 (25.6)	38.3 (32.4, 44.4)	
**Perception of disease severity**			
Mild/Moderate	125 (61.6)	40.6 (33.9, 46.7)	0.358
Severe	78 (38.4)	39.7 (31.7, 45.7)	
**Knowledge of TB**			
Poor	22 (10.8)	37.5 (30.1, 41.9)	0.189
Good	181 (89.2)	40.6 (32.8, 46.4)	
**Alcohol intake**			
Yes	85 (41.9)	41.7 (33.6, 46.4)	0.269
No	118 (58.1)	38.4 (32.1, 45.7)	
**Cigarette smoking**			
Yes	50 (24.6)	41.1 (33.2, 46.1)	0.614
No	153 (75.4)	39.4 (32.2, 46.7)	
**Length of treatment(months)**			
≤ 3 months	94 (46.3)	40.3 (33.2, 46.1)	0.835
>3 months	109 (53.7)	39.4 (32.2, 46.4)	
**Barrier to TB treatment**			
Yes	36 (17.7)	38.6 (31.3, 43.1)	0.114
No	167 (82.3)	40.6 (32.8, 46.7)	

NB: HRQoL = Health-related quality of life, IQR = Interquatile range, TB = tuberculosis

Bivariate analyses revealed several factors significantly associated with total HRQoL. Higher median HRQoL scores were observed among participants aged < 30 years (42.8, IQR 34.2–58.5) compared with those aged 30–59 years (39.7, IQR 32.2–45.6) and ≥ 60 years (37.2, IQR 30.6–41.7; *p* = 0.015). Males reported significantly higher HRQoL than females (41.7, IQR 33.9–46.7 vs. 37.8, IQR 30.6–44.4; *p* = 0.025). Being single (41.7, IQR 33.8–56.0), having a tertiary education (43.9, IQR 33.9–60.6), and being HIV-negative (41.1, IQR 33.3–46.7) were also associated with significantly better HRQoL (*p* < 0.05; Table 1).

Partial correlation analyses showed strong positive associations between medication adherence and all HRQoL domains before controlling for future orientation: physical (*r* = 0.373, *p* < 0.001), social (*r* = 0.535, *p* < 0.001), emotional (*r* = 0.377, *p* < 0.001), functional (*r* = 0.410, *p* < 0.001), and spiritual (*r* = 0.476, *p* < 0.001). After controlling for future orientation, the correlations weakened considerably, particularly for the emotional domain (*r* = 0.093, *p* = 0.188). In contrast, the correlation coefficients for the physical (*r* = 0.180, *p* = 0.010), social (*r* = 0.365, *p* < 0.001), functional (*r* = 0.265, *p* < 0.001), and spiritual (*r* = 0.284, *p* < 0.001) domains were reduced (Table 2). Adherent participants had higher median HRQoL scores across all domains than non-adherent participants, and 79.3% of participants were classified as adherent (all *p* < 0.001; Table 3).

**Table 2:** Partial correlation between adherence and the domains of HRQoL before and after controlling for future orientation

**Correlation before controlling for future orientation**							
**Variables**	**Adherence r**, ***p***	**Physical HRQoL *r***, ***p***	**Social HRQoL *r***, ***p***	**Emotional HRQoL *r***, ***p***	**Functional HRQoL *r***, ***p***	**Spiritual HRQoL *r***, ***p***	**Future Orientation *r***, ***p***
Adherence	1						
Physical HRQoL	0.373, < 0.001	1					
Social HRQoL	0.535, < 0.001	0.491, < 0.001	1				
Emotional HRQoL	0.377, < 0.001	0.485, < 0.001	0.459, < 0.001	1			
Functional HRQoL	0.410, < 0.001	0.440, < 0.001	0.507, < 0.001	0.220, 0.002	1		
Spiritual HRQoL	0.476, < 0.001	0.287, < 0.001	0.477, < 0.001	0.278, < 0.001	0.403, < 0.001	1	
Future Orientation	0.647, < 0.001	0.381, < 0.001	0.442, < 0.001	0.487, < 0.001	0.340, < 0.001	0.434, < 0.001	1
**Correlation after controlling for future orientation**							
**Variables**	**Adherence *r***, ***p***	**Physical HRQoL *r***, ***p***	**Social HRQoL *r***, ***p***	**Emotional HRQoL *r***, ***p***	**Functional HRQoL *r***, ***p***	**Spiritual HRQoL *r***, ***p***	
Adherence	1						
Physical HRQoL	0.180, 0.010	1					
Social HRQoL	0.365, < 0.001	0.389, < 0.001	1				
Emotional HRQoL	0.093, 0.188	0.370, < 0.001	0.311, < 0.001	1			
Functional HRQoL	0.265, < 0.001	0.357, < 0.001	0.423, < 0.001	0.067, 0.345	1		
Spiritual HRQoL	0.284, < 0.001	0.146, 0.039	0.353, < 0.001	0.084, 0.236	0.301, < 0.001	1	

NB: FO = Future orientation, HRQoL = Health related quality of life, r = correlation coefficient

**Table 3:** Association between adherence and health-related quality of life

Variable	Adherence *n* = 161	Non-adherence *n* = 42	*p*
Domains (median, IQR)			
Physical	27.9 (20.6, 34.6)	11.8 (8.4, 14.7)	< 0.001
Social	67.9 (53.6, 78.6)	35.7 (27.7, 42.9)	< 0.001
Emotional	38.6 (29.6, 48.9)	18.2 (13.6, 25.6)	< 0.001
Functional	50.0 (39.3, 71.4)	30.4 (20.5, 42.9)	< 0.001
Spiritual	83.3 (70.8, 91.7)	50.0 (33.3, 66.7)	< 0.001
Total	42.2 (36.9, 48.1)	25.6 (23.3, 29.4)	< 0.001

NB: IQR = Interquatile range. Medication adherence was reported by 161 (79.3%)

MMAS^®^ 2006 used with permission www.moriskyscale.com

A hierarchical multiple regression analysis was conducted to examine predictors of HRQoL and to assess whether future orientation moderates the association between medication adherence and HRQoL (Table 4). Predictors were entered in three blocks: (1) sociodemographic and clinical covariates (age, gender, marital status, educational status, HIV status); (2) medication adherence and future orientation; and (3) the medication adherence × future orientation interaction term.

**Table 4:** Hierarchical multiple regression analysis on HRQoL

Variable	Step 1 B, p-value		Step 2 B, p-value		Step 3 B, p-value	
Age	-0.186	0.014	-0.159	0.011	-0.146	0.019
Gender	-0.158	0.017	-0.125	0.023	-0.102	0.064
Marital status	-0.122	0.106	-0.055	0.380	-0.052	0.403
Educational status	0.299	<0.001	0.172	0.002	0.183	0.001
HIV status	0.125	0.059	0.091	0.099	0.084	0.123
Adherence	0.316	<0.001	0.450	<0.001		
Future orientation	0.262	<0.001	0.293	<0.001		
Adherence x future orientation	0.201	0.018				
F	10.677	<0.001	23.607	<0.001	21.877	0.001
R^2^	0.220	0.469	0.485			
Adjusted R^2^	0.200	0.449	0.463			
ΔR²	0.220	0.249	0.016			

In Step 1, the covariates explained 22.0% of the variance in HRQoL (R² = 0.220, adjusted R² = 0.200, F(5, 197) = 10.677, *p* < 0.001). Significant predictors included age (B = -0.186, *p* = 0.014), gender (B = -0.158, *p* = 0.017), and educational status (B = 0.299, *p* < 0.001), with HIV status marginally significant (B = 0.125, *p* = 0.059). Step 2 added adherence and future orientation, significantly improving the model (ΔR² = 0.249, R² = 0.469, adjusted R² = 0.449, F(7, 195) = 23.607, *p* < 0.001). Both adherence (B = 0.316, *p* < 0.001) and future orientation (B = 0.262, *p* < 0.001) were strong positive predictors. In Step 3, the inclusion of the interaction term (medication adherence × future orientation) resulted in a statistically significant improvement in model fit (ΔR² = 0.016, *p* = 0.018), yielding a final model with R² = 0.485, adjusted R² = 0.463, F (8, 194) = 21.877, *p* < 0.001. The interaction was significant (B = 0.201, *p* = 0.018), indicating that future orientation moderates the association between medication adherence and HRQoL. Although the interaction term was statistically significant, it explained a relatively small proportion of additional variance in HRQoL (ΔR² = 0.016), indicating a modest moderation effect. In the final model, adherence (B = 0.450, *p* < 0.001), future orientation (B = 0.293, *p* < 0.001), age (B = -0.146, *p* = 0.019), and educational status (B = 0.183, *p* = 0.001) remained significant predictors. Gender became marginally non-significant (B = -0.102, *p* = 0.064), while HIV status and marital status were not significant.

**Fig. 1:**
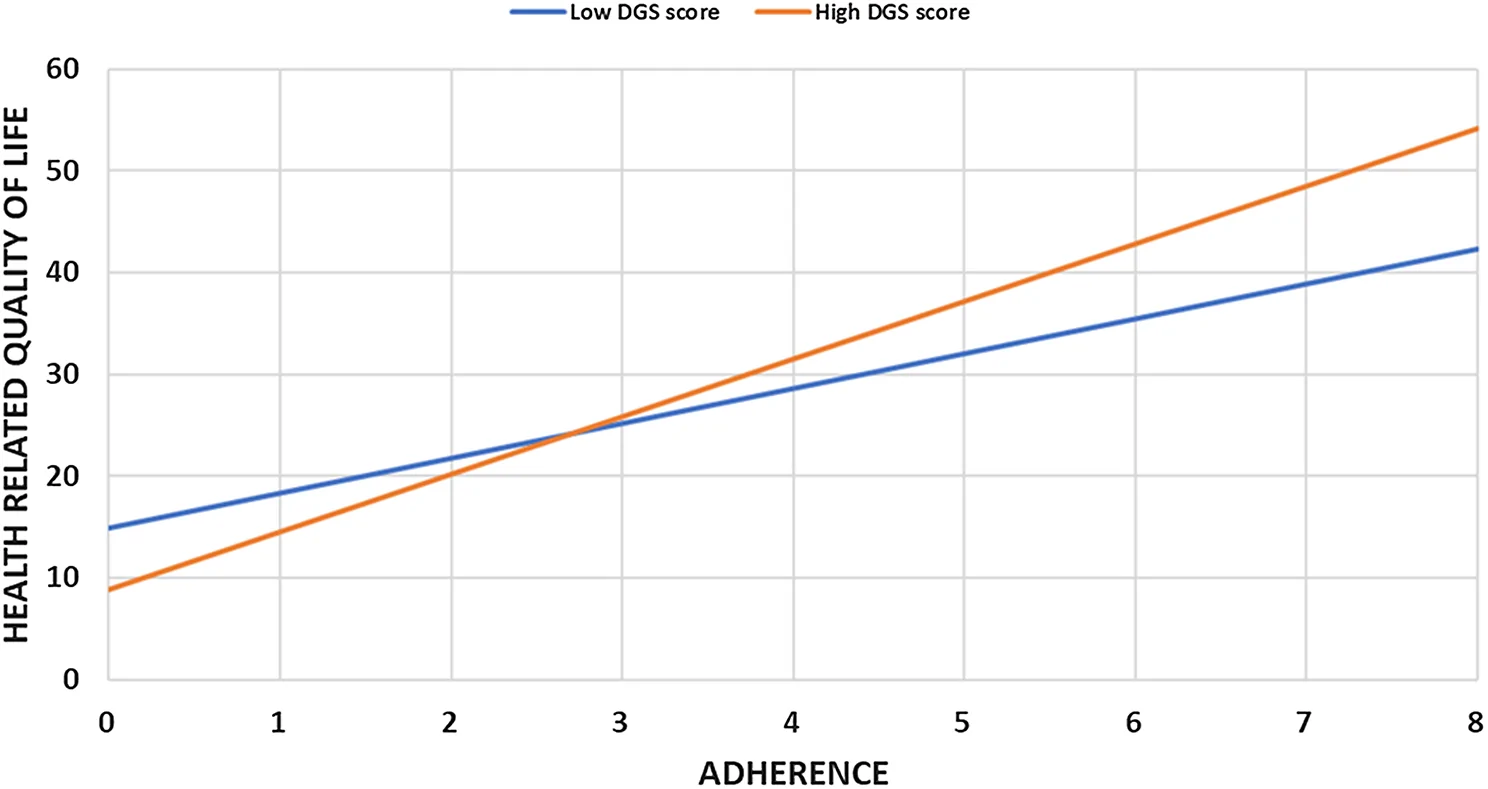
Simple slope plot of the interaction between adherence and future orientation on health-related quality of life

Simple slopes analysis (Table 5) showed that the positive association between medication adherence and HRQoL was stronger at high levels of future orientation (+ 1 SD = 46.96; β ≈ 0.644, t = 5.297, *p* < 0.001) compared with lower levels (− 1 SD = 32.89; β ≈ 0.390, t = 5.371, *p* < 0.001). The regression lines crossed at an adherence score of approximately 2.70, such that, for very low adherence scores, predicted HRQoL was slightly higher for low future orientation; this reversed and increased substantially with higher adherence, particularly beyond the sample mean (Adherence ≈ 6.2) (Fig. 1).

**Table 5:** Slope analysis showing the impact of adherence on HRQoL at low and high future orientation

**Adherence**	**β**	**t**	**p**
Low future orientation	0.390	5.371	<0.001
High future orientation	0.664	5.297	<0.001

## Discussion

This study examined whether future orientation moderates the association between medication adherence and HRQoL among PwDR-TB in Lagos, Nigeria. The findings indicate that both medication adherence and future orientation were positively associated with HRQoL, and future orientation was statistically associated with the strength of the association between medication adherence and HRQoL. Specifically, the positive association between medication adherence and HRQoL was stronger among individuals with higher levels of future orientation. However, the interaction effect accounted for a relatively small proportion of variance in HRQoL, indicating that the moderation effect, although statistically significant, was modest in magnitude. These findings reflect associations rather than directional or causal effects because of the study design. This may suggest that while future orientation is an important psychological factor, it represents only one component within a broader set of determinants influencing patient-reported outcomes.

HRQoL among PwDR-TB is influenced by a complex interplay of clinical, psychological, and socioeconomic factors [24]. In the present study, older age, female sex, and lower educational attainment were associated with poorer HRQoL, consistent with findings from other high-burden settings, including Nigeria, Ethiopia, and Nepal, where structural inequalities adversely affect patient-reported outcomes [25–27]. Medication adherence was also positively associated with HRQoL, corroborating findings from studies in Nepal, India, and Indonesia [3, 28, 29]. Given the prolonged duration of DR-TB treatment, medication toxicity, and associated socioeconomic challenges, maintaining adherence is likely to be an important correlate of patient-centred outcomes [30, 31]. However, inconsistent findings from other settings suggest that the relationship between adherence and HRQoL may be influenced by additional psychological, clinical, and contextual factors [4, 32].

The present study extends the literature by suggesting that future orientation may modify the association between medication adherence and HRQoL. Nevertheless, the modest interaction effect indicates that future orientation accounted for only a small proportion of the variability in HRQoL beyond adherence and other covariates. Consequently, the clinical importance of this moderation should be interpreted cautiously. Broader influences, including treatment burden, socioeconomic disadvantage, mental health, and health-system constraints, are likely to contribute more substantially to HRQoL than future orientation alone.

Several mechanisms could potentially explain the observed association. Individuals with stronger future orientation may be more likely to perceive treatment-related difficulties as worthwhile investments in future health, maintain engagement with treatment despite delayed benefits, or adopt more consistent health-promoting behaviours [33, 34]. These pathways are particularly relevant in DR-TB, where sustained commitment is required despite delayed clinical benefits [35]. These interpretations are biologically and psychologically plausible and align with behavioural theory. However, because the present study was cross-sectional, these mechanisms remain speculative and require confirmation in longitudinal and experimental studies that can establish temporal relationships.

From a programmatic perspective, the findings provide preliminary evidence that future orientation may be relevant to understanding patient-reported outcomes among PwDR-TB. However, the modest magnitude of the moderation effect and the cross-sectional design mean that the results should not be interpreted as evidence that interventions targeting future orientation will improve adherence or HRQoL. Instead, these findings support further investigation of future-oriented cognition within longitudinal and intervention studies. Should future research confirm these associations, behavioural approaches such as goal-setting, motivational interviewing, or cognitive behavioural strategies could be evaluated as potential complements to established adherence-support interventions, alongside broader efforts to address structural and health-system barriers to DR-TB care.

## Limitations

Several limitations should be acknowledged. First, the cross-sectional design precludes establishing the temporal ordering of medication adherence, future orientation, and HRQoL. Consequently, the observed moderation should be interpreted as a statistical association rather than evidence that future orientation causally modifies the relationship between adherence and HRQoL. Reverse causation is also possible, as individuals with better HRQoL may be more likely to adhere to treatment or report a stronger future orientation. Second, the sampling strategy and recruitment of participants from treatment centres in Lagos may limit the generalizability of the findings to other settings. In addition, the exclusion of severely ill patients and the inclusion of only English-speaking participants may have introduced selection bias, resulting in an overestimation of HRQoL. Third, medication adherence, future orientation, and HRQoL were all assessed using self-reported questionnaires. Self-reported adherence, in particular, may be susceptible to recall error and social desirability bias, with participants potentially overstating their adherence to align with perceived expectations. Furthermore, because all key variables were measured using similar self-report instruments during a single assessment, common method bias may have inflated the observed associations and interaction effects. Although validated instruments with good internal consistency were used, measurement bias cannot be excluded. Finally, although several important socio-demographic and clinical covariates were included in the analyses, unmeasured factors such as depression, resilience, stigma, treatment-related adverse effects, and economic hardship may have influenced both future orientation and HRQoL. Future studies should employ longitudinal designs to clarify temporal relationships, incorporate objective measures of medication adherence (e.g., pill counts, pharmacy refill records, electronic monitoring, or biochemical assays), and use multi-method approaches to reduce common method bias and better evaluate the role of future orientation in patient-centred outcomes among people with drug-resistant tuberculosis. Although the overall sample size was adequate for the primary analyses, interaction effects typically require larger samples to detect small effect sizes. Therefore, the moderation analysis should be regarded as exploratory and warrants confirmation in larger prospective studies.

## Conclusion

Future orientation was found to moderate the association between medication adherence and HRQoL among people with PwDR-TB in Lagos, Nigeria, although the magnitude of this interaction was modest. Better medication adherence was associated with higher HRQoL, and this association appears stronger among individuals with higher levels of future orientation. These findings suggest that future orientation may be an important psychological factor associated with patient-reported outcomes in DR-TB. However, because of the cross-sectional design, the observed associations should not be interpreted as causal or directional. Longitudinal and intervention studies are needed to determine whether strengthening future-oriented cognition influences medication adherence, HRQoL, or their relationship over time.

## Supplementary Information

Below is the link to the electronic supplementary material.

Supplementary Material 1

Supplementary Material 2

Supplementary Material 3

Supplementary Material 4

## Data Availability

The datasets used and/or analysed during the current study are available from the corresponding author on reasonable request.

## Abbreviations

DR-TB: Drug-resistant tuberculosis
FACIT TB: The Functional Assessment of Chronic Illness Therapy– Tuberculosis
HRQoL: Health-related quality of life
HIV/AIDS: Human immunodeficiency syndrome/ Acquired Immune Deficiency Syndrome
MMAS-8: Morisky Medication Adherence Scale
TB: Tuberculosis
PwDR-TB: People with drug-resistant tuberculosis
