# Supplementary material for: Future orientation moderates the relationship between medication adherence and health-related quality of life among people with drug-resistant tuberculosis in Lagos, Nigeria: a cross-sectional study
Source: BMC Infect Dis. 2026 Aug 6;26:1942. doi: 10.1186/s12879-026-14163-z (PMC13737692; doi:10.1186/s12879-026-14163-z)
Supplement: Supplementary file 1 [file 12879_2026_14163_MOESM1_ESM.pdf]

Functional Assessment of Chronic Illness Therapy-Tuberculosis (FACIT-TB) FACIT-TB Scale

| Items | Yes | No |
|---|---|---|
| Domain: Physical Well-being | | |
| 1. I feel ill | [__] | [__] |
| 2. I get tired easily | [__] | [__] |
| 3. I have a lack of energy | [__] | [__] |
| 4. I have pain | [__] | [__] |
| 5. I feel weak all over | [__] | [__] |
| 6. I feel fatigued | [__] | [__] |
| 7. I have been short of breath | [__] | [__] |
| 8. I have nausea "a sense of vomiting outbreak" | [__] | [__] |
| 9. Because of my physical condition, I have trouble meeting the needs of my family | [__] | [__] |
| 10. I am bothered by fever (episode of high body temperature) | [__] | [__] |
| 11. I am forced to spend time in bed | [__] | [__] |
| 12. I have discomfort or pain in my stomach area | [__] | [__] |
| 13. I have had itching | [__] | [__] |
| 14. I have a loss of appetite | [__] | [__] |
| 15. I have been coughing | [__] | [__] |
| 16. I am bothered by side effects of treatment | [__] | [__] |
| 17. Dust worsens my symptoms | [__] | [__] |
| Domain: Social and economic well-being | | |
| 18. I feel close to my friends | [__] | [__] |
| 19. I get emotional support from my family | [__] | [__] |
| 20. I am satisfied with family communication about my illness | [__] | [__] |
| 21. My family has accepted my illness | [__] | [__] |
| 22. I feel close to my partner (or the person who is my main support) | [__] | [__] |
| 23. I get support from my friends | [__] | [__] |
| 24. My physical condition and/or medical treatment cause me financial difficulties | [__] | [__] |
| Domain: Emotional well-being/Stigma of having TB | | |
| 25. I worry that my condition will get worse | [__] | [__] |
| 26. I worry about dying | [__] | [__] |
| 27. I am concerned about what the future holds for me | [__] | [__] |
| 28. I am embarrassed by my illness | [__] | [__] |
| 29. It is hard to tell other people about my disease | [__] | [__] |
| 30. I am losing hope in the fight against my illness | [__] | [__] |
| 31. I am bothered by change in weight | [__] | [__] |
| 32. I worry about spreading my infection | [__] | [__] |
| 33. I feel nervous | [__] | [__] |
| 34. I feel sad | [__] | [__] |
| 35. I am satisfied with how I am coping with my illness | [__] | [__] |

| | | |
|---|---|---|
| Domain: Functional well-being | | |
| 36. I am content with the quality of my life right now | [__] | [__] |
| 37. My work (include work at home) is fulfilling | [__] | [__] |
| 38. I am able to work (include work at home) | [__] | [__] |
| 39. I am able to enjoy life | [__] | [__] |
| 40. I am enjoying the things I usually do for fun | [__] | [__] |
| 41. I have accepted my illness | [__] | [__] |
| 42. I am sleeping well | [__] | [__] |
| Domain: Spiritual well-being | | |
| 43. I find strength in my faith or spiritual belief | [__] | [__] |
| 44. My illness has strengthened my faith or spiritual belief | [__] | [__] |
| 45. My life is still productive | [__] | [__] |
