# Supplementary material for: Future orientation moderates the relationship between medication adherence and health-related quality of life among people with drug-resistant tuberculosis in Lagos, Nigeria: a cross-sectional study
Source: BMC Infect Dis. 2026 Aug 6;26:1942. doi: 10.1186/s12879-026-14163-z (PMC13737692; doi:10.1186/s12879-026-14163-z)
Supplement: Supplementary file 2 [file 12879_2026_14163_MOESM2_ESM.pdf]

Morisky 8-item Medication adherence scale

Items	**Yes**	**No**
1. Do you sometimes forget to take your TB pills?	[__]	[__]
2. Over the past two weeks, were there any days when you did not take your TB medicine?	[__]	[__]
3. Have you ever cut back or stopped taking your medication without telling your doctor because you felt worse when you took it?	[__]	[__]
4. When you travel or leave home, do you sometimes forget to bring along your medications?	[__]	[__]
5. Did you take your TB medicine yesterday?	[__]	[__]
6. When you feel like your TB symptoms are under control, do you sometimes stop taking your medicine?	[__]	[__]
7. Taking medication every day is a real inconvenience for some people. Do you ever feel hassled about sticking to your TB treatment plan?	[__]	[__]
8. Are there any additional challenges that you face in trying to complete your TB treatment?	[__]	[__]

The MMAS-8 Scale, content, name, and trademarks are protected by US copyright and trademark laws. Permission for use of the scale and its coding is required. A license agreement is available from MMAR, LLC., www.moriskyscale.com.

The MORISKY SCALE (© 2007 Donald E. Morisky)
