# Supplementary material for: Future orientation moderates the relationship between medication adherence and health-related quality of life among people with drug-resistant tuberculosis in Lagos, Nigeria: a cross-sectional study
Source: BMC Infect Dis. 2026 Aug 6;26:1942. doi: 10.1186/s12879-026-14163-z (PMC13737692; doi:10.1186/s12879-026-14163-z)
Supplement: Supplementary file 3 [file 12879_2026_14163_MOESM3_ESM.pdf]

Deferment of Gratification Scale (DGS)

For the next set of statements, please think about whether you agree or disagree with the statement. For example, if the statement is extremely uncharacteristic of you, you may choose “Strongly Disagree.” On the other hand, if the statement is extremely characteristic of you, you may choose “Strongly Agree.” Use the options in the middle if you fall somewhere in between.

	Items	**STRONGLY AGREE**	**AGREE**	**NOT SURE**	**DISAGREE**	**STRONGLY DISAGREE**
DGS 1	You are good in saving your money so you can buy bigger things at a later time					
DGS 2	Relief from stress in the present is more important to you than having good health in the future					
DGS 3	As a child I saved any pocket money that I had					
DGS 4	When I am at the supermarket I buy more things than I planned for					
DGS 5	I am constantly without money					
DGS 6	I agree with the philosophy eat drinK and enjoy yourself for tomorrow we may all be dead					
DGS 7	I describe myself as acting without thinking for my own good					
DGS 8	I often think it is important to wait and think things over before deciding					
DGS 9	I like spending my money immediately I get it					
DGS 10	It is hard for me to avoid losing my temper when someone makes me angry					
DGS 11	It is easy for me to be patient when waiting for things most times					
DGS 12	You think you are good at planning for things ahead					
