# Supplementary material for: Future orientation moderates the relationship between medication adherence and health-related quality of life among people with drug-resistant tuberculosis in Lagos, Nigeria: a cross-sectional study
Source: BMC Infect Dis. 2026 Aug 6;26:1942. doi: 10.1186/s12879-026-14163-z (PMC13737692; doi:10.1186/s12879-026-14163-z)
Supplement: Supplementary file 4 [file 12879_2026_14163_MOESM4_ESM.pdf]

Morisky Medication adherence research LLC

LETTER OF PERMISSION

Certificate Number: 5175-8387-0577-7395-2255
5-May-2026

To Whom It May Concern:

This letter confirms that permission is hereby granted to Dr Adejumo Olusola from Durban University of Technology, to use the Morisky Medication Adherence Scale, 8-item version (MMAS-8), for the purposes of the academic research study entitled:

"Future orientation moderates the relationship between medication adherence and health-related quality of life among people with drug-resistant tuberculosis in Lagos, Nigeria"

This permission is granted for use in data collection, analysis, academic dissemination, and publication in all formats, including print and digital media, and is compatible with publication under a Creative Commons Attribution 4.0 International (CC BY 4.0) open access license, provided that all conditions outlined below are strictly followed.

The original validated wording, structure, and scoring of the MMAS-8 must be maintained without modification. The Morisky Medication Adherence Research, LLC (MMAR, LLC), as the authorized copyright holder, must be appropriately credited in all publications and outputs.

This permission is limited, non-transferable, and applies solely to the study described above. It does not confer ownership or broader licensing rights and may not be extended to third parties without prior written authorization. Use of the MMAS-4 remains subject to the applicable End User License Agreement. This permission may be revoked if the stated terms are violated.

Intellectual Property Notice

The Morisky Medication Adherence Scales (MMAS-4 and MMAS-8) and their derivatives are protected intellectual property of Morisky Medication Adherence Research, LLC (MMAR, LLC). All rights are reserved worldwide. No alterations, adaptations, or derivative works are permitted without prior written consent. Unauthorized use, reproduction, or distribution may constitute infringement and will be subject to enforcement under applicable law.

"xxxx."

The requirements to use this scale are to cite the following reference in the document:

1. **Krousel-Wood M, Islam T, Webber LS, Re RN, Morisky DE, Muntner P. New medication adherence scale versus pharmacy fill rates in seniors with hypertension. Am J Manag Care. 2009 Jan;15(1):59-66. PMID: 19146365; PMCID: PMC2728593.**

i. The following footnote is required in all articles, presentations, web postings, reports and submitted manuscripts, in the Acknowledgment Section of manuscripts submitted for publication:

MMAS® 2006 used with permission www.moriskyscale.com

Philip Morisky (May 5, 2026 09:41:29 PDT)

Philip Morisky, MBA
Chief Operations
Morisky Medication Adherence Research, LLC
5-May-2026

permission letter_Dr Adejumo Olusola MMAS-8

Final Audit Report 2026-05-05

Created:	2026-05-05
By:	Philip Morisky (philm715@icloud.com)
Status:	Signed
Transaction ID:	CBJCHBCAABAA345LvmP5EWDVKFFi6QYcUNUaleYx4HtF

"permission letter_Dr Adejumo Olusola MMAS-8" History

Document created by Philip Morisky (philm715@icloud.com)
2026-05-05 - 4:37:38 PM GMT- IP address: 97.93.62.244

Document emailed to Philip Morisky (philm715@icloud.com) for signature
2026-05-05 - 4:37:42 PM GMT

Email viewed by Philip Morisky (philm715@icloud.com)
2026-05-05 - 4:41:01 PM GMT- IP address: 146.75.146.1

Document e-signed by Philip Morisky (philm715@icloud.com)
Signature Date: 2026-05-05 - 4:41:29 PM GMT - Time Source: server- IP address: 97.93.62.244

Agreement completed.
2026-05-05 - 4:41:29 PM GMT
